# Bacterial Profiling and Dynamic Succession Analysis of *Phlebopus portentosus* Casing Soil Using MiSeq Sequencing

**DOI:** 10.3389/fmicb.2019.01927

**Published:** 2019-08-23

**Authors:** Rui-Heng Yang, Da-Peng Bao, Ting Guo, Yan Li, Guang-Yan Ji, Kai-Ping Ji, Qi Tan

**Affiliations:** ^1^Key Laboratory of Edible Fungal Resources and Utilization (South), National Engineering Research Center of Edible Fungi, Shanghai Academy of Agricultural Sciences, Institute of Edible Fungi, Shanghai, China; ^2^Key Laboratory of Agricultural Genetics and Breeding of Shanghai, National Engineering Research Center of Edible Fungi, Shanghai Academy of Agricultural Sciences, Institute of Edible Fungi, Shanghai, China; ^3^Hongzhen Agricultural Science and Technology Co. Ltd., Jinghong, China

**Keywords:** casing soil, decontamination, bacterial diversity, dynamics, MiSeq

## Abstract

*Phlebopusportentosus* (Berk. and Broome) Boedijin is a popular edible mushroom found in China and Thailand. To date, *P. portentosus* is the only species in the order Boletales that can be successfully cultivated worldwide. The use of a casing layer or casing soil overlaying the substrate is a crucial step in the production of this mushroom. In this study, bacterial profiling and dynamic succession analyses of casing soil during the cultivation of *P. portentosus* were performed. One hundred and fifty samples were collected, and MiSeq sequencing of the V3-V4 region of the 16S rRNA gene was conducted. After performing a decontamination procedure, only 38 samples were retained, including 6 casing soil-originated samples (OS), 6 casing soil samples (FHCS) and 5 upper substrate samples (FHCU) from the period of complete colonization by mycelia; 6 casing soil samples (PCS) and 5 upper substrate samples (PCU) from the primordium period; and 6 casing soil samples (FCS) and 4 upper substrate samples (FCU) from fruit body period. The results revealed that bacterial diversity increased sharply from the hyphal to the primordium stage and then decreased during harvesting. The non-metric multidimensional scaling (NMDS) ordination and analysis of similarities (ANOSIM) analysis suggested that the community composition during different stages was significantly different in casing soil. The most abundant phyla in all of the samples were *Proteobacteria*, *Chloroflexi*, *Acidobacteria*, *Actinobacteria*, *Saccharibacteria*, and *Bacteroidetes*. *Burkholderia* was the most abundant genus in all the samples except the OS samples. The relative abundance of *Burkholderia* in the FHCS samples (55.79%) decreased to 35.14% in the PCS samples and then increased to 45.60% in the FCS samples. The abundances of *Acidobacterium*, *Rhizobium*, *Acidisphaera*, *Bradyrhizobium*, and *Bacillus* increased from the FHCS to PCS samples. The linear discriminant analysis (LDA) effect size (LEfSe) suggested that *Acidobacterium* and *Acidisphaera* are micromarkers for PCS, whereas *Bradyrhizobium*, *Roseiarcus*, and *Pseudolabrys* were associated with fruit body stages. The network analyses resulted in 23 edges, including 4 negative and 19 positive edges. Extensive mutualistic interactions may occur among casing soil bacteria. Furthermore, these bacteria play important roles in mycelial elongation, primordium formations, and the production of increased yields.

## Introduction

*Phlebopus portentosus* (Berk. and Broome) Boedijin (also known as tropical black bolete) belongs to the order Boletales and is a popular edible mushroom in both China and Thailand (Zhang et al., 2017). *P. portentosus* is widely distributed in Asia, America, Oceania, China, Thailand, Indonesia, Sri Lanka, Vietnam, Australia, New Zealand, Brazil, Mexico, and other tropical regions (Heinemann and Rammeloo, 1982; Segedin, 1987; Watling, 2001; Sanmee et al., 2003; Bandala et al., 2004; Ji, 2007). *P. portentosus* was previously reported to form a mutualistic relationship with the roots of some plants, characterizing it as an ectomycorrhizal (ECM) fungus (Kumla et al., 2016). However, unlike its close relatives in the family Boletaceae, such as *Boletus edulis*, *P. portentosus* can be artificially cultivated and produce sporocarps in artificial substrate *in vitro* (Ji, 2007; Ji et al., 2011; Kumla et al., 2015). Up to now, the cultivation of *P. portentosus* has been highly industrialized, as in the *Agaricus* mushroom industry, which has resulted in daily production of 2 tons of *P. portentosus*. To date, this is the only species in the Boletales which can be successfully cultivated worldwide.

For the commercial cultivation of *P. portentosus*, a crucial step in the production of this mushroom is the addition of a casing layer or casing soil overlaying the substrate (Kumla et al., 2015). Many mushrooms need a casing layer to induce the formation of fruiting bodies or increase yields, e.g., *Agaricus bisporus*, *Agaricus blazei*, *Copyinds comatus*, *Grifola frondosa*, *Ganoderma lucidum*, *Stropharia rugosoannulata*, and so on (Martos et al., 2017; Kertesz and Thai, 2018; Carrasco et al., 2019b). Casing soil-associated bacteria and fungi are known to influence the growth and development of *Agaricus bisporus* and other commercial mushrooms (Kertesz and Thai, 2018). Kertesz and Thai (2018) summarized the roles that these bacteria and fungi play, including the conversion of the lignocellulose substrate into nutrient-rich compost for mushroom growth; interactions with the fungal mycelia during hyphal elongation and proliferation through the substrate; and the induction of primordial fruiting body formation (Segedin, 1987). For example, *Pseudomonas*, *Bacillus* and other bacteria can increase mycelial growth, primordial formation, and yield in some species of the genera *Agaricus* and *Pleurotus* (Park and Agnihotri, 1969; Rainey, 1991; Cho et al., 2003; Cai et al., 2009; Young et al., 2012; Zarenejad et al., 2012; Chen et al., 2013; Rossouw and Korsten, 2017). Furthermore, the bioactive factors in the casing soil may promote fruiting body formation in *P. portentosus*.

Several studies have revealed that some bacteria isolated from the casing soil of *P. portentosus* have a significant effect on mycelial growth and fruiting body formation (Ji et al., 2011; Liu et al., 2011, 2012; Wang et al., 2013; Wu et al., 2013). *Bacillus* species from the phylum Firmicutes and *Brevibacterium* species from the phylum *Actinobacteria*, as well as other *Actinobacteria* strains, have been shown to play important roles in promoting mycelial elongation, fruiting body formation, and increased individual weight (Ji et al., 2011; Liu et al., 2011, 2012; Wang et al., 2013; Wu et al., 2013). The results also suggested that there was a complicated bacterial network influencing the development of *P. portentosus.* To characterize the bacterial structure and dynamics in compost and casing soil in other mushrooms, molecular methods and next-generation sequencing have been widely used at an unprecedented scale, e.g., PCR-DGGE, PLFA, 454 pyrosequencing and MiSeq methodologies (Szekely et al., 2009; De Gannes et al., 2013; Kertesz et al., 2016). However, only cultivation methods have been used to assess the bacterial community present in the casing soil used for *P. portentosus* (Ji et al., 2011; Liu et al., 2011, 2012; Wang et al., 2013; Wu et al., 2013; Kertesz and Thai, 2018). The use of the MiSeq next-generation sequencing platform to detect the profiles and dynamic succession bacteria in casing soil for *P. portentosus* may provide insights into the interactions between casing soil-associated bacteria and *P. portentosus.*

In this study, an examination of the bacterial structures and dynamics associated with casing soil at different stages during the cultivation of *P. portentosus* was conducted using MiSeq sequencing of 16S ribosomal DNA (rDNA). As a unique cultivated species of the order Boletales, *P. portentosus* may be another useful model to study the mechanisms of casing soil to influence the growth of mushrooms.

## Materials and Methods

### Artificial Cultivation of *Phlebopus portentosus*

**Figure 1:**
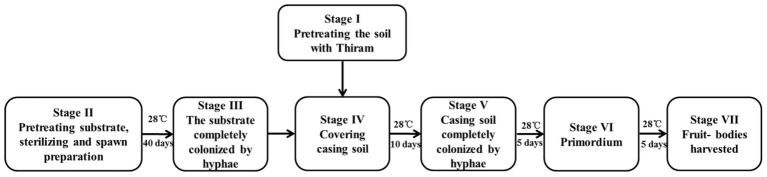
The *P. portentosus* cultivation process.

The *P. portentosus* strain 17026, which was isolated from a natural mature fruiting body, was provided by Hongzhen Agricultural Science and Technology Co. Ltd. The strain was maintained on modified PDA medium at 30°C in the dark. All the primary and secondary spawn preparation and culturing of the mushrooms was conducted at Hongzhen Agricultural Science and Technology Co. Ltd. The substrate was primarily composed of sawdust, starch, and soil. The substrate was placed into 1,100-ml culture bottles (8.5 cm diameter and 14.0 cm height) and sterilized at 121°C for 2 h (S, stage II). The sterilized bottles were inoculated with the spawn of *P. portentosus* strain 17026 and incubated at 28°C for approximately 40 days to obtain completely colonized hyphae (H, stage III), after which the substrate-hyphae in culture bottles were covered with 2 cm of casing soil (stage IV). After continuous incubation for 10 days, the casing soil was full of fungal hyphae (FH, stage V). The primordia of the fruit bodies appeared after 5 days (P, stage VI), at which time the fruit bodies were harvested (F, stage VII). The entire cultivation process was shown in Figure 1.

The soil used for the casing layers was collected from banana fields (banana was the most dominant plant species) near the company (21.68 N 100.72 E). The total nitrogen (TN) concentrations of soil samples were measured using a Kjeltec TM2300 azotometer (FOSS, Sweden); total phosphorus (TP) was estimated using the colorimetric method with ammonium molybdate in hydrochloric acid; and total potassium (TK) was determined using a flame photometer (Gao et al., 2019). Humic acids (HAs) were extracted using the classic alkali/acid fractionation procedure (Valdrighi et al., 1996). Subsequently, the soil was pretreated with Thiram (stage I) to avoid fungal contamination and then used as the casing soil. There were three treatments performed for this study for stage IV, (1) normal casing soil (90 samples); (2) no covering casing soil (24 samples); and (3) sterilized casing soil (36 samples).

### Sampling

Each culture bottle was divided into two sections, an upper section and a lower section. During each stage, samples were collected from the upper and lower sections of each bottle, with six repetitions conducted. In stage I, only the casing soil was collected; in stages II and III, six upper section samples and six lower section samples were collected; in stages V and VI, six casing soil samples, six upper and six lower section samples were collected; and in stage VII, six casing soil samples, six upper section samples, six lower section samples, and six fruit bodies were collected. For treatments 2 and 3, samples were collected used the same strategies from the stages V and VI. Thus, a total of 150 samples were collected in this study.

### DNA Extraction, Polymerase Chain Reaction Amplification, and MiSeq Sequencing

DNA extractions from the substrate and casing soil were conducted using a commercial PowerSoil DNA Isolation Kit (MoBio Laboratories, Carlsbad, CA, USA) according to the manufacturer’s protocol. The primer pair 338F/806R was used to amplify the V3-V4 hypervariable region of the bacterial 16S rRNA gene (Zhang et al., 2018). A 6 bp-barcode was added to the 5′ end of the forward and reverse primers for discriminating different samples. Polymerase chain reaction (PCR) amplification was performed in a 50 μl volume containing 25 μl of 2 × PCR MIX, 2 μl of template DNA, 1 μl of barcoded primers, and 21 μl of H_2_O. The following thermocycling conditions were used for PCR amplification: 94°C for 2 min, followed by 30 cycles of 94°C for 30 s, 57°C for 30 s, and 72°C for 45 s, with a final extension at 72°C for 10 min (Langenheder and Szekely, 2011). Three PCR repetitions for each sample were performed to offset PCR bias. The mixed PCR products were purified using a QIAquick Gel Extraction kits (QIAGEN, Germany) and quantified *via* Real Time PCR. Illumina MiSeq sequencing was performed at Personalbio Company (Shanghai, China).

### Data Processing

The paired-end reads were assembled into one scaffold using Mothur v.1.40.0 (Schloss et al., 2009) with a minimum overlap of 10 bp. The assembled reads were filtered and removed according to the criterions: length < 200 bp; average quality score ≤ 30; contained ambiguous base calls and did not exactly align with primer sequences and barcode tags. The qualifying reads were sorted into different samples based on their unique barcodes. Importantly, a non-standard pipeline suggested by Łukasik et al., 2017 was to filter out suspected contaminant genotypes or noise from the dataset. The samples from stage II were used as the negative controls. The procedure consisted of four steps. In the first step, the unique sequences (results from Mothur using the script unique.seqs), unless having a relative abundance in at least one experimental library that was at least 10 times its maximum relative abundance in any of the negative controls, were excluded from the dataset. In the second step, to avoid the retention of low-abundance sequences in samples, the unique sequences that did not account for at least 0.1% of at least one experimental library were removed. In the third step, samples where the number of the reads filtered over 75% of the starting number of reads were discarded, as these samples were deemed to be too highly contaminated. In the fourth step, the steps above were repeated. Finally, only high-quality, uncontaminated reads were retained.

### Operational Taxonomic Unit Clustering, Taxonomy, Alpha Diversity, Beta Diversity, and Statistics

The reads in the dataset were clustered into operational taxonomic units (OTUs) using QIIME at a 97% sequence similarity (Caporaso et al., 2010). The microbial diversity and richness were evaluated using the Shannon and ACE indices, both of which were tested by *t*-tests. The distance matrices of community composition (Hellinger-transformation of the OTU data) of casing soil were generated by calculating dissimilarities using the Bray-Curtis method. To further visualize the community composition dissimilarities in each stage, non-metric multidimensional scaling (NMDS) together with analysis of similarities (ANOSIM) was conducted using the metaMDS and anosim command in the vegan package (version 2.4.1). Taxonomic classifications of the effective sequences were carried out using the RDP Classifier (Wang et al., 2007). As the relative abundance of bacterial compositions at the phylum, class, and genus levels did not satisfy the normality of distribution and homogeneity of variance after the square root and log transformation, a nonparametric Kruskal-Wallis test was used. Linear discriminant analysis effect size (LEfSe, LDA = 4.0) was used to identify bacterial markers from the different stages. To construct the co-occurrence network of the core bacteria from the different stages, bivariate correlation analysis was performed, and the network was generated using Cytoscape (version 3.2.1). Networks were visualized using a prefuse force directed layout in which the nodes represent the bacterial genera and the edges represent the correlation (negative-blue; positive-red). All of the reads were submitted to the NCBI Sequence Read Archive under the accession numbers SRR9018053-SRR9018090.

## Results

### *Phlebopus portentosus* Cultivation and the Physicochemical Properties of Casing Soil

**Figure 2:**
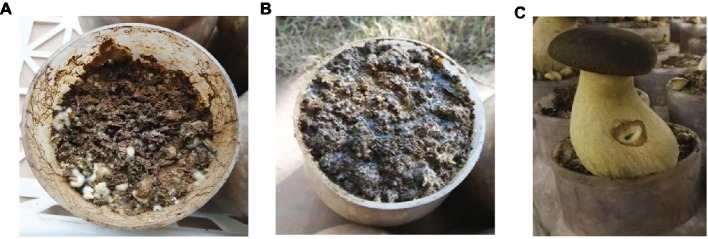
The cultivation of *P. portentosus* in the three treatments. **(A)** No covering casing soil; **(B)** sterilized casing soil; **(C)** normal covering casing soil.

After a 60-day incubation period, different results were obtained from the three treatments. The samples with no casing soil and sterilized casing soil did not form fruiting bodies (Figures 2A,B), whereas the formation of healthy fruit bodies was induced by the normal casing soil (Figure 2C). The average weight of the individual fruiting body was 126 g.

The total N, P, K, and organic matter contents in the normal and sterilized casing soil were not different significantly (Supplementary Table S1).

### Sequencing and Filtering Information

The DNA extractions and V3-V4 16S rRNA gene region amplifications were successful in all the samples except one from stage VII. Finally, 5,094,718 assembled high-quality reads were generated from the 149 samples. The number of reads across all the samples ranged from 24,308 to 72,653 (Supplementary Table S2), and the average number of reads in each sample was 34,192 (Supplementary Table S2). After the first decontamination step, only 682,922 reads were retained, accounting for 13.40% of the total reads (Supplementary Table S2), and the number of reads across all of the samples ranged from 47 to 17,858 (Supplementary Table S1). One hundred and eleven samples retained less than 25% of the starting number of reads after the filtering steps and were removed from subsequent analyses. Finally, 424,200 high-quality, contamination-free reads were retained in 38 samples (Supplementary Table S1). The 38 samples included 24 casing soil samples from stages I (OS), V (FHCS), VI (PCS), and VII (FCS), 5 upper substrate samples from stage V (FHCU), 5 upper substrate samples from stage VI (PCU), and 4 upper substrate samples from stage VII (FCU) (Supplementary Table S1). The results also showed that samples from the lower substrate, samples taken before covering the soil, and fruit bodies were all filtered out. Because the number of reads in each sample was highly uneven, 7,096 reads per sample were randomly selected to balance the reads in each sample for subsequent analyses.

### Diversity and Richness in Different Samples

**Figure 3:**
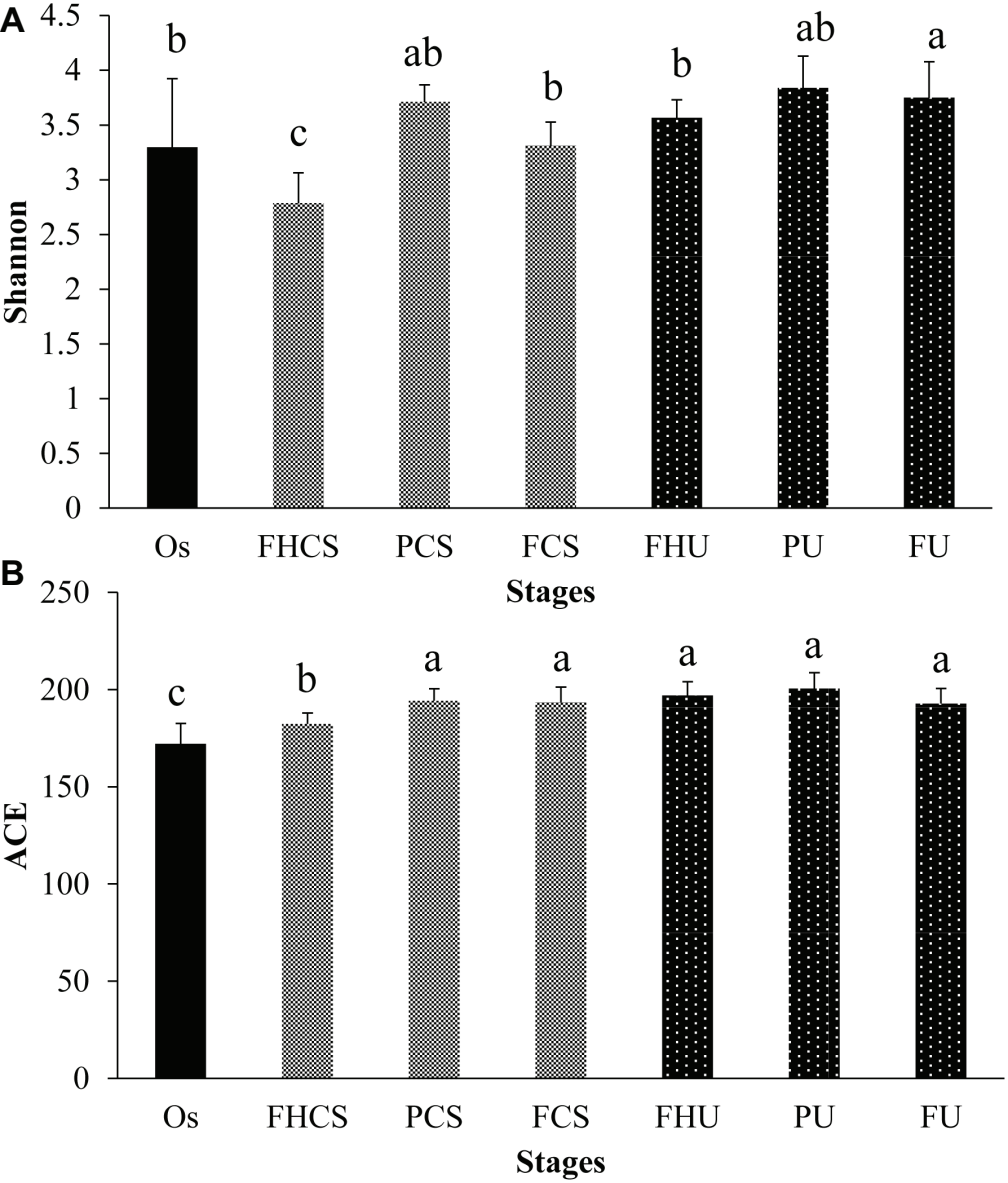
Microbial diversity and richness. The Shannon and ACE indices were calculated at a depth of 7,096 reads in each sample. The bars indicate the mean; the error bars denote standard deviation; and the lowercase letters above the bars indicate significant differences. FHCS, casing soil from stage V (casing soil completely colonized by hyphae); PCS, casing soil from stage VI (primordial stage); FCS, casing soil from stage VII (fruiting body stage); FHU, upper portion of the substrates from stage V; PU, upper portion of the substrates from stage VI; FU, upper portion of the substrates from stage VII. **(A)** Shannon, **(B)** ACE.

Two hundred and thirty-one OTUs were clustered in all the samples. The microbial diversity and richness were calculated by assessing the OTU composition in each sample. In the casing soil samples, the OTU diversity varied significantly during the growth of *P. portentosus*, where the Shannon index was 3.30 in the original casing soil samples (OS, stage I) and decreased to 2.79 in the FHCS samples (stage V) (Figure 3A). A sharp increasing in the Shannon index value (3.71) was observed in the PCS stage (stage VII) (Figure 3A), and then decreased to 3.31 before the fruit bodies were harvested (stage FCS). The OTU richness continually increased from 172 in the OS (stage I) to 182 in stage the FHCS (stage V). For the samples from upper substrate, the Shannon index increased and became stable in stage V (Figure 3B), whereas the ACE index values in the different upper substrate samples were nearly identical (Figure 3B).

### Comparisons of Community Composition in Different Stages

**Figure 4:**
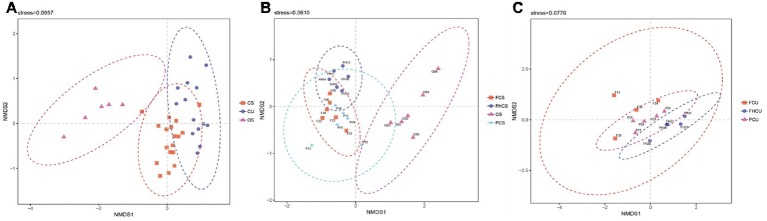
Non-metric multidimensional scaling (NMDS) ordination of the bacterial community composition at different stages of *P. portentosus* cultivation. Ellipses in the plots denote 95% confidence intervals for the centroids of the CS, CU, and OS samples. FHCS: casing soil from stage V (casing soil completely colonized by hyphae); PCS, casing soil from stage VI (primordial stage); FCS, casing soil from stage VII (fruiting body stage); FHU, upper portion of the substrates from stage V; PU, upper portion of the substrates from stage VI; FU, upper portion of the substrates from stage VII. **(A)** NMDS for CS, CU and OS, **(B)** NMDS for FCS, FHCS, OS and PCS, **(C)** NMDS for FCU, FHCU and PCU.

The results of the non-metric multidimensional scaling (NMDS) ordination analysis suggested that the community composition at different stages was significantly different. The bacterial community was different between the OS and CS and CU samples (Figure 4A). The fungus influenced the bacterial structures in the casing soil samples from the different stages (Figure 4B), demonstrating that a folded linearity fluctuate of bacterial structure occurred during *P. portentosus* development. There were no differences between samples from the upper substrate (*p* > 0.05, Figure 4C). The results of the ANOSIM analysis were in agreement with those of the NMDS analysis.

### Bacterial Compositions in Different Samples

**Figure 5:**
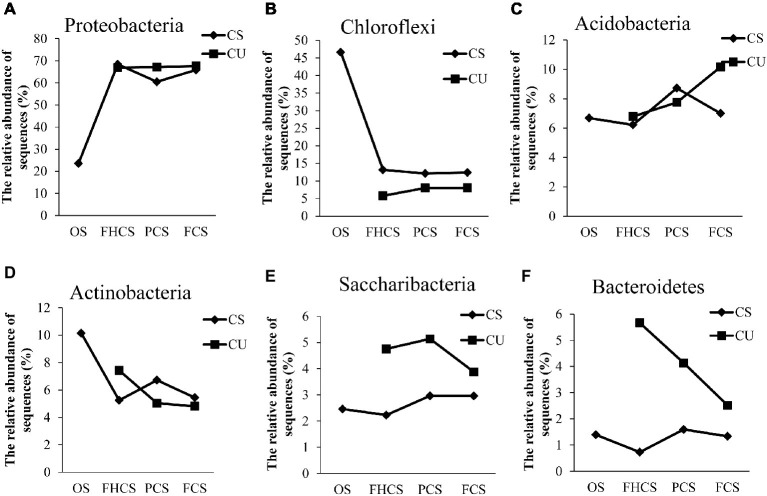
The dynamics of some phyla in the casing soil and upper substrate samples during the development of *P. portentosus*. **(A)** *Proteobacteria*; **(B)** *Chloroflexi*; **(C)** *Acidobacteria*; **(D)** *Actinobacteria*; **(E)** *Saccharibacteria*; **(F)** *Bacteroidetes*. CS, samples from casing soil; CU, samples from the upper substrate. FHCS, casing soil from stage V (casing soil completely colonized by hyphae); PCS, casing soil from stage VI (primordial stage); FCS, casing soil from stage VII (fruiting body stage); FHU, upper portion of the substrates from stage V; PU, upper portion of the substrates stage VI; FU, upper part of the substrates stage VII.

At the phylum level, 97.6% of the total sequences were assigned to 16 known phyla. The most abundant phyla in all the samples were *Proteobacteria, Chloroflexi, Acidobacteria, Actinobacteria, Saccharibacteria*, and *Bacteroidetes*, accounting for 60.00, 15.18, 7.62, 6.40, 3.48, and 2.48% of the total phyla, respectively (Supplementary Figure S1). The relative abundance of *Proteobacteria* sharply increased from the casing soil OS samples (23.55%) to the FHCS samples (68.39%). Subsequently, the percentage of *Proteobacteria* decreased to 60.49% in the PCS samples and increased to 65.84% in the FCS samples (Figure 5A). The observed changes in the abundances of *Acidobacteria*, *Actinobacteria*, and *Bacteroidetes* were the same, with an initial decrease from the OS to FHCS samples followed by an increase from the FHCS to PCS samples and a subsequent further decrease from the PCS to the FCS samples (Figures 5C,F,D). The relative abundance of Chloroflexi sharply decreased from 46.61% in the OS samples to 13.15% in the FHCS samples (Figure 5B), whereas that of *Saccharibacteria* exhibited a slow decrease from the OS to the FHCS samples before increasing to a plateau in stage VI (PCS) (Figure 5E).

The relative abundances of *Proteobacteria* and *Chloroflexi* in upper substrate nearly did not vary throughout the process (Figures 5A,B). In contrast, a continued increase in the abundance of *Acidobacteria* was observed, from 6.78 to 10.16% in the FHCU and FCU samples, respectively (Figures 5A,B). The relative abundance of *Actinobacteria* and *Bacteroidetes* continued decreasing (Figures 5D,F). The percentage of *Saccharibacteria* increased from the OS samples (4.75%) to the FHCS samples (5.14%) and then decreased to 3.88% in the stage VI (PCS) samples (Figure 5E).

**Figure 6:**
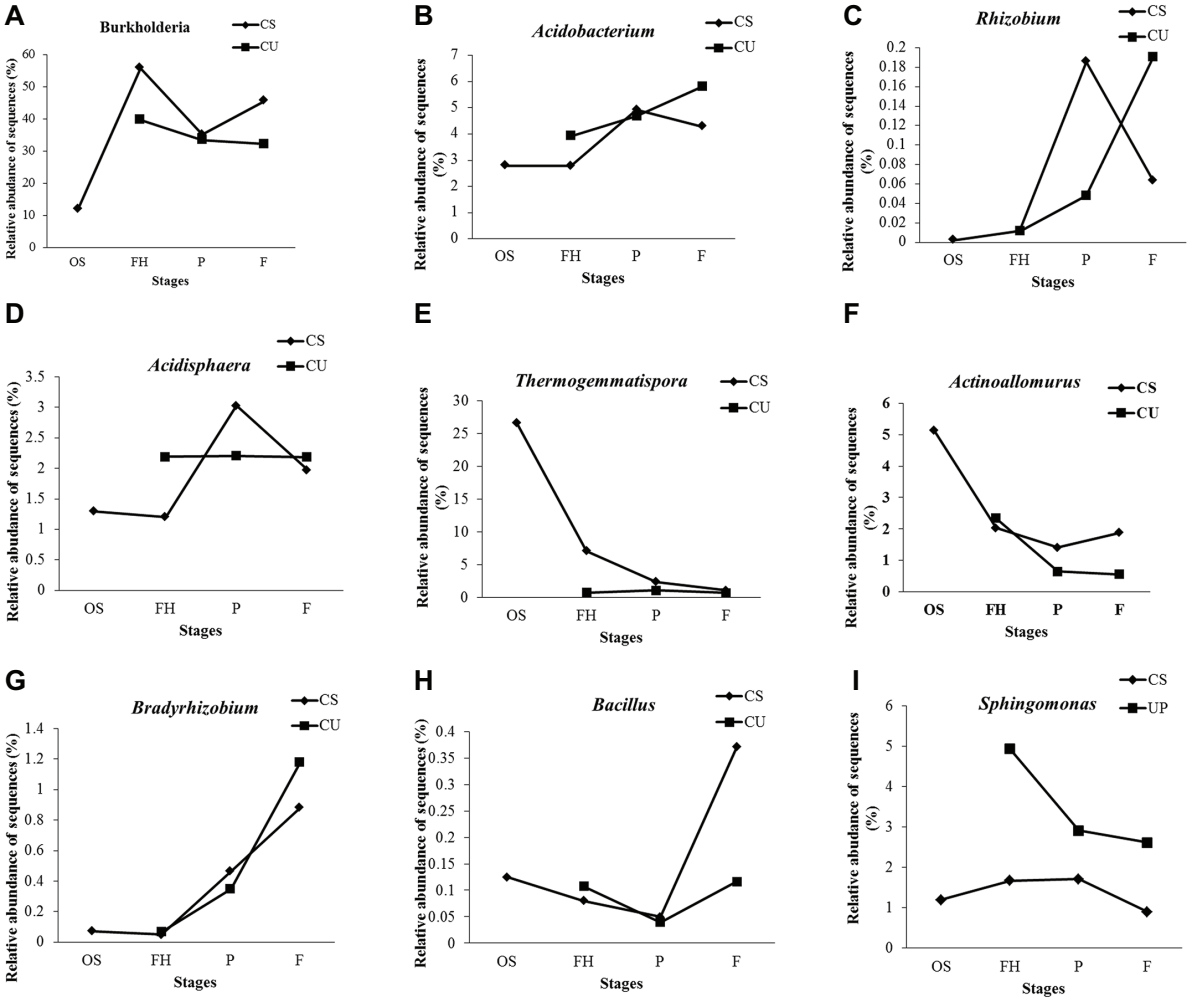
The dynamics of some genera in the casing soil and upper substrate samples during the development of *P. portentosus*. **(A)** *Burkholderia*; **(B)** *Acidobacterium*; **(C)** *Acidobacteria*; **(D)** *Acidisphaera*; **(E)** *Thermogemmatispora*; **(F)** *Actinoallomurus*; **(G)** *Bradyrhizobium*; **(H)** *Bacillus*; **(I)** *Sphingomonas*. CS, samples from the casing soil; CU, samples from the upper substrate.

At the genus level, 76 genera were detected and annotated in all of the samples, 53.10% of which belonged to *Burkholderia* (36.26%), *Thermogemmatispora* (5.70%), *Acidobacterium* (4.17%), *Bryobacter* (2.43%), *Sphingomonas* (2.27%), and *Rhizomicrobium* (2.27%) (Supplementary Figure S2). The relative abundance of *Burkholderia* was higher in the FH samples (55.79%) than in the OS samples (11.95%) but decreased to 35.14% in the PCS samples before increasing to 45.60% in the FCS samples (Figure 6A). The same increasing trends of *Acidobacterium*, *Rhizobium*, and *Acidisphaera* were observed from the FHCS to the PCS samples, while the same decreasing tendencies were observed from the PCS to the FCS samples (Figures 6B–D). The relative abundance of *Thermogemmatispora* exhibited a continual decrease from 26.68 to 1.10% in the OS and FCS samples, respectively (Figure 6E), as did that of *Actinoallomurus*, which decreased from 5.13% in the OS samples to 1.41 and 1.88% in the PCS and FCS samples, respectively (Figure 6F). The abundance of *Bradyrhizobium* increased to 0.47% in the PCS samples and 0.88% in FCS samples, which was 100 times higher than that observed in the OS and FHCS samples (Figure 6G). A sharp increase in the abundance of *Bacillus* was also observed, from 0.049% in PCS samples to 0.37% in FCS (Figure 6H). A higher abundance of *Sphingomonas* was detected in the FHCS (1.67%) and PCS (1.71%) samples than in the OS (1.19%) and FCS (0.89%) samples (Figure 6I).

Except for *Bacillus* (Figure 6H), the relative abundances of the eight other detected genera continually increased, decreased, or remained stable in the upper substrate (CU) sample throughout the entire process of *P. portentosus* cultivation. The abundances of *Acidisphaera* and *Thermogemmatispora* remained stable during the growth process (Figures 6D,E).

### Microbiota Biomarkers in the Different Stages or Samples

**Figure 7:**
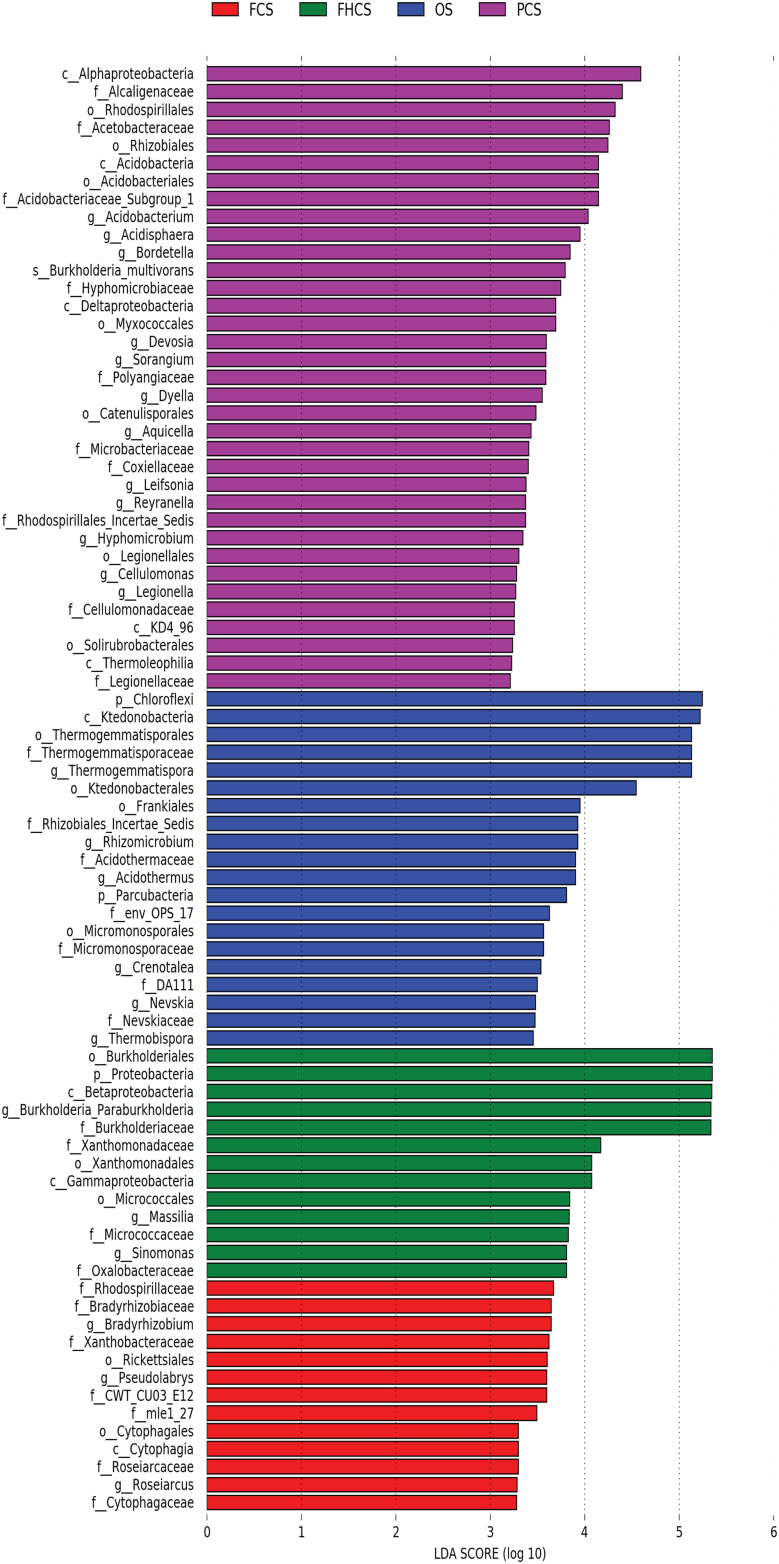
Identification of crucial bacteria associated with different stages. LEfSe reports the taxonomic representation of statistically and biologically consistent differences between the OS, FHCS, PCS, and FCS bacterial communities. OS, original casing soil; FHCS, casing soil completed with colonized hyphae; PCS, casing soil during the primordial period; FCS, casing soil during the fruiting body period.

The linear discriminant analysis (LDA) effect size method was used to identify the greatest differences in taxa among the different casing soil samples. The differential features were identified based on the OTU compositions. LEfSe identified 35, 20, 13, and 13 clades or taxa in the PCS, OS, FHCS, and FCS samples, respectively (Figure 7). At the genus level, *Thermogemmatispora*, *Crenotalea*, *Acidothermus*, *Nevskia*, *Rhizomicrobium*, and *Thermobispora* were significantly enriched in OS samples; *Burkholderia*, *Massilia*, and *Sinomonas* were significantly enriched in the FHCS samples; *Sorangium*, *Acidobacterium*, *Burkholderia*, *Bordetella*, *Leifsonia*, *Reyranella*, *Hyphomicrobium*, *Aquicella*, *Legionella*, *Dyella*, *Devosia*, and *Acidisphaera* were significantly enriched in the PCS samples; and *Bradyrhizobium*, *Roseiarcus*, and *Pseudolabrys* were the most significantly enriched in the fruiting body stages (FCS).

### Topological Consistency Between Operational Taxonomic Units Across Networks

**Figure 8:**
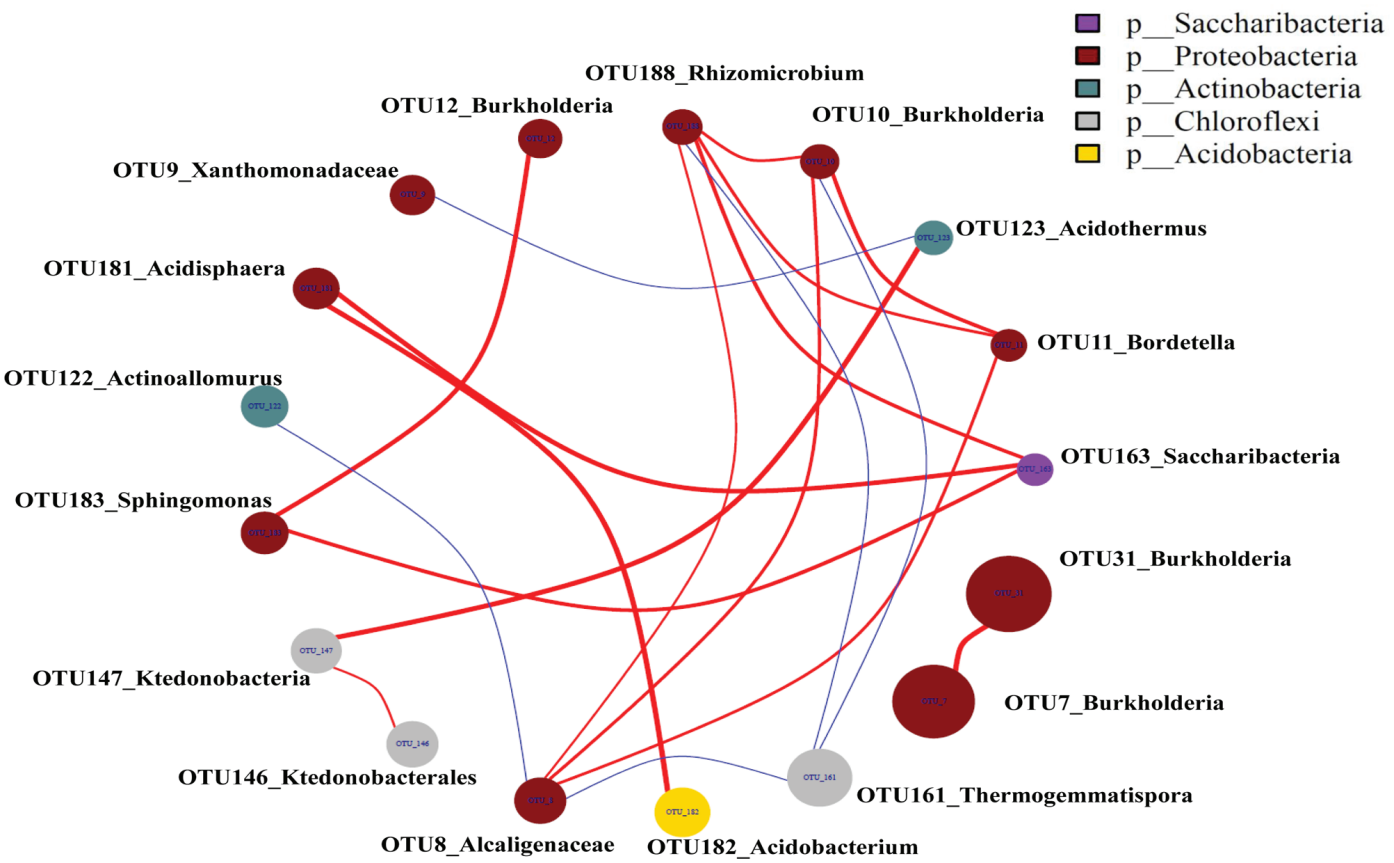
Microbial networks in the casing soil and upper substrate samples based on the top 20 OTUs. The red color edges were positive associations, and the blue edges were negative associations (*p* < 0.05). A connection indicates a strong positive Spearman’s correlation coefficient if *p* > 0.75 and a negative Spearman’s correlation coefficient occurred if *p* < −0.75.

The top 20 OTUs were present at least in 2 networks that resulted in 23 edges, including 4 negative and 19 positive edges (Figure 8), revealing that 19 pairs of OTUs had consistently positive correlations and 4 pairs of OTUs had consistently negative correlations. The negative correlations were only observed between OTU123_Acidothermus and OTU9_Xanthomonadaceae, OTU10_Burkholderia and OTU161_Thermogemmatispora, OTU188_Rhizomicrobium and OTU161_Thermogemmatispora, OTU8_Alcaligenaceae and OTU161_Thermogemmatispora, and OTU8_Alcaligenaceae and OTU122_Actinoallomurus.

## Discussion

In this study, an investigation of the dynamics of microbiota in casing soil during the development of *P. portentosus* was conducted based on 16S rRNA sequencing using the MiSeq platform. The results revealed that the bacterial community varied across the *P. portentosus* cultivation process. Interestingly, there was also a dynamic change in the relative abundance of some specific taxa that were either positively or negatively associated with mycelial elongation, primordial stimulation, and fruiting body formation.

An important decontamination procedure was used in this study to filter suspected contaminating sequences generated from the sequencing, PCR, or negative samples. It was previously reported that bacterial DNA is present in reagents and in the laboratory environment that can alter the microbial communities of samples, particularly those with a relatively low template concentration (Salter et al., 2014). These suspicious artifacts derived from one sample may affect the amplification process for other samples (Kircher et al., 2012). Thus, Łukasik et al. (2017) recommended a non-standard analysis pipeline based on the use of some negative controls to filter out the suspected contaminant genotypes or sequences from the MiSeq-generated dataset. In our study, although the sterilized samples used as negative controls were considered to be DNA- or bacteria-free, PCR amplification and MiSeq sequencing were successful. These results suggested that bacterial DNA was still present in these samples, although no active bacteria survived. These DNA templates used for PCR may have influenced the estimations of bacterial communities. In addition, the identified rare sequences were also excluded in this study. This step is crucial since these sequences may exhibit an increased influence under some reweighting schemes, and it may be beneficial to delineate their true presence from simple noise effects (Pylro et al., 2014). We also recommended that a decontaminating procedure was conducted in other investigations of bacterial communities.

Like *Agaricus*, the presence of a diverse bacterial population in casing layers has been shown to be essential for primordial formation, as fruiting did not occur with sterilized casing layers (Eger, 1963; Hayes et al., 1969; Park and Agnihotri, 1969; Eger, 1972). The production of the mushroom fruiting bodies were induced when isolated bacteria from casing soils returned to the sterile casing layer over axenically grown cultures of *A. bisporus* in compost, which revealed that bacteria was the essential factor for fruiting body formation (Eger, 1972). In this study, the primordia or fruit bodies of *P. portentosus* could not form when no casing soil and sterilized casing soil were used (Figure 2A). Some physicochemical properties in the normal and sterilized soil were similar, indicating that biotic factors may play important roles in the formation of fruiting bodies, e.g., bacteria and fungi. The Shannon index values of bacteria increased sharply from the hyphal stage to the primordium stage and then decreased during the harvesting stage in the process of *P. portentosus* cultivation. These results were in agreement with those from *Agaricus*, where the bacterial diversity in casing soil increased dramatically after the initiation of the first primordia but decreased during later flushes (Cai et al., 2009; Partanen et al., 2010). The formation of primordia and fruit bodies in *Agaricus* was previously shown to be reinitiated by the addition of bacterial inoculums or adsorptive carbon-based materials. These results revealed that the stimulatory role of the bacteria involved the removal of an inhibitor of primordial formation, including 1-octen-3-ol, which controls the early differentiation of vegetative hyphae to multicellular knots (Hayes et al., 1969; Rainey, 1991; Noble et al., 2003, 2009; Eastwood et al., 2013; Colauto et al., 2016). And some ACC deaminase-producing bacteria lower the levels of ethylene produced by *Agaricus* to stimulate primordial formation (Wood and Hammond, 1977; Chen et al., 2013; Zhang et al., 2016). However, these results were not confirmed in *P. portentosus*, for which the stimulatory mechanisms need to be further studied.

Members of the genus *Burkholderia* were the major bacteria observed to be associated with *P. portentosus* in this study. This finding corroborated previous studies that also detected this taxon in association with ECM root tips and surrounding soil of *Suillus luteus*, *Tricholoma matsutake*, *Leccinum extremiorientale*, and so on (Ryota et al., 2012; Li et al., 2016; Oh et al., 2016; Qin et al., 2016; Nishino et al., 2018). *Burkholderia* species have emerged common and potentially important bacteria associated with plants, animals, and especially fungi (Santos et al., 2001; Stoyanova et al., 2007), with some of these bacteria having been shown to be useful in promoting plant growth (Stoyanova et al., 2007). No *Burkholderia* living within the hyphae of *Rhizopus* resulted in losing the abilities to produce reproductive sporangia or spores (Partida-Martinez et al., 2007), which revealed that *Burkholderia* can affect fungal development and spore production. Members of the genus *Burkholderia* have been shown to have the ability to fix nitrogen associated with root tips. Whether *in vitro* nitrogen is an important interaction between *Burkholderia* and *P. portentosus* requires further study. However, some *Burkholderia* are the most common pathogens detected in other mushroom and can inhibit mushroom formation, including mycelial elongation and primordial formation in *Agaricus* and *Pleurotus* (Mignucci et al., 2000; Largeteau and Savoie, 2010). Another surprising finding was that no *Pseudomonas* species were detected, as these bacteria are accepted to play influential roles in the development of *Pleurotus* and *Agaricus* mushroom fruiting bodies (Noble et al., 2003; Kertesz and Thai, 2018). Meanwhile, the most abundant genus detected in the casing soil of *Agaricus bisporus* was *Flavobacterium* (Carrasco et al., 2019a). The differences in the interactions between fungi and bacteria may be associated with specific ecotypes (ECM and saprophytic fungi) and genetic backgrounds (secretions: enzymes or product of metabolisms).

The relative abundances of *Acidobacterium*, *Bradyrhizobium*, *Rhizobium*, and *Acidisphaera* increased along with the development of *P. portentosus*, which may be associated with the formation of *P. portentosus* primordia. *Rhizobium* in the casing soil of *A. bisporus* was previously shown to increase pinning (Park and Agnihotri, 1969), and *Acidobacterium* and *Bradyrhizobium* were enriched in the active shiro of *Suillus granulatus* and *Tricholoma matsutake* during the fruiting process in pine (Ryota et al., 2012; Zhu et al., 2013; Zhang et al., 2015). *Acidobacterium* and *Acidisphaera* were identified as micro-markers for primordial formation, while *Bradyrhizobium* was the signature genus for fruiting body formation in *P. portentosus*. The relative abundance of *Bacillus* only increased after primordial formation, suggesting that *Bacillus* plays important roles in the process of *P. portentosus* fruiting body maturation. It was previously demonstrated that *Brevibacterium* and *Bacillus* could promote mycelial elongation, fruiting body formation, and increased individual weight in *P. portentosus* (Liu et al., 2012) and other mushrooms (Zhu et al., 2013; Ekinci and Dursun, 2014; Kertesz and Thai, 2018). However, some other bacterial genera may also be associated with the development of *P. portentosus*, e.g., *Sorangium*, *Bordetella*, *Reyranella*, *Hyphomicrobium*, *Aquicella*, *Legionella*, *Dyella*, *Devosia*, *Roseiarcus*, and *Pseudolabrys*, as extensive mutualistic interactions may occur among casing soil bacteria (Figure 8; Shi et al., 2016).

The cultivation of *P. portentosus* is similar to that of *Lentinula* grown in fully sterilized substrate, as the enzymes secreted by mycelia are crucial to the growth and crop performance of *Lentinula* (Philippoussis et al., 2003). Although the addition of specific bacteria could potentially stimulate either mushroom growth or fruiting body formation, this possibility has not been well investigated in *P. portentosus*. The high bacterial diversity present in casing soil plays important roles in mushroom mycelial elongation, primordial formation, and increased yields. Thus, the recovery of the bacteria from casing soil used to grow *P. portentosus* as well as non-casing soil used in the cultivated process should be the focus of future investigations.

## Data Availability

All the sequences generated for this study have been submitted to NCBI under the accession numbers of SRR9018053-SRR9018090.

## Author Contributions

R-HY, D-PB, K-PJ, and QT contributed in the conception and design of the study. TG, YL, and G-YJ performed the sampling. R-HY performed the analysis and wrote the manuscript draft. All authors contributed to manuscript revision and approved the final version.

### Conflict of Interest Statement

K-PJ and G-YJ are employed by the Hongzhen Agricultural Science and Technology Co. Ltd.

The remaining authors declare that the research was conducted in the absence of any commercial or financial relationships that could be construed as a potential conflict of interest.

## Supplementary Material

The supplementary material for this article is available online at: https://www.frontiersin.org/articles/10.3389/fmicb.2019.01927/full#supplementary-material
