# Supplementary material for: Bacterial Profiling and Dynamic Succession Analysis of Phlebopus portentosus Casing Soil Using MiSeq Sequencing
Source: Front Microbiol. 2019 Aug 23;10:1927. doi: 10.3389/fmicb.2019.01927 (PMC6716355; doi:10.3389/fmicb.2019.01927)
Supplement: Supplementary file 1 [file Data_Sheet_1.docx]

Table S1. Total organic matter, N, P and K content in the different soil samples

|  | OM (g/kg) | TP (mg/g) | TN (mg/g) | TK (mg/g) |
| --- | --- | --- | --- | --- |
| normal casing soil | 92.87±9.78a | 0.26±0.00a | 1.04±0.03a | 0.11±0.00a |
| sterilized casing soil | 92.82±4.03a | 0.25±0.00a | 1.02±0.04a | 0.11±0.00a |

Note: OM: organic matter; TP: total phosphorus; TN: total nitrogen; TK: total potassium.

*Same letters within each column indicate no significant difference among the treatments (at P < 0.05) according to LSD test.

Table S2. Sequencing and filtering information

| Stages | Samples | pretreatment of casing soil | Sample type | The number of initial reads in each sample | The number of reads in each sample after step 1 | The number of reads in each sample after step2 | The relative abudance of reads in each sample after step3 (%) | The number of reads in each sample after step4 | Step 4: Relative percentage of the starting number of reads (%) | The number of final reads used in each sample | Final decision |
| --- | --- | --- | --- | --- | --- | --- | --- | --- | --- | --- | --- |
| Stage II | S11 | Normal | upper | 31390 | 124 | 110 | 0 | 0 | 0 | - | deleted |
| Stage II | S12 | Normal | upper | 35371 | 354 | 335 | 1 | 0 | 0 | - | deleted |
| Stage II | S13 | Normal | upper | 36486 | 96 | 70 | 0 | 0 | 0 | - | deleted |
| Stage II | S14 | Normal | upper | 33104 | 120 | 104 | 0 | 0 | 0 | - | deleted |
| Stage II | S15 | Normal | upper | 35358 | 158 | 139 | 0 | 0 | 0 | - | deleted |
| Stage II | S16 | Normal | upper | 65105 | 89 | 80 | 0 | 0 | 0 | - | deleted |
| Stage II | S21 | Normal | Lower | 36735 | 100 | 79 | 0 | 0 | 0 | - | deleted |
| Stage II | S22 | Normal | Lower | 35397 | 65 | 64 | 0 | 0 | 0 | - | deleted |
| Stage II | S23 | Normal | Lower | 39193 | 47 | 35 | 0 | 0 | 0 | - | deleted |
| Stage II | S24 | Normal | Lower | 35262 | 55 | 49 | 0 | 0 | 0 | - | deleted |
| Stage II | S25 | Normal | Lower | 43709 | 127 | 96 | 0 | 0 | 0 | - | deleted |
| Stage II | S26 | Normal | Lower | 37049 | 88 | 78 | 0 | 0 | 0 | - | deleted |
| Stage III | H11 | Normal | upper | 36740 | 280 | 150 | 0 | 0 | 0 | - | deleted |
| Stage III | H12 | Normal | upper | 33632 | 374 | 172 | 1 | 0 | 0 | - | deleted |
| Stage III | H13 | Normal | upper | 37296 | 498 | 319 | 1 | 0 | 0 | - | deleted |
| Stage III | H14 | Normal | upper | 32650 | 432 | 192 | 1 | 0 | 0 | - | deleted |
| Stage III | H15 | Normal | upper | 31161 | 238 | 142 | 0 | 0 | 0 | - | deleted |
| Stage III | H16 | Normal | upper | 30484 | 69 | 61 | 0 | 0 | 0 | - | deleted |
| Stage III | H21 | Normal | Lower | 62793 | 142 | 114 | 0 | 0 | 0 | - | deleted |
| Stage III | H22 | Normal | Lower | 32019 | 278 | 174 | 1 | 0 | 0 | - | deleted |
| Stage III | H23 | Normal | Lower | 29378 | 504 | 418 | 1 | 0 | 0 | - | deleted |
| Stage III | H24 | Normal | Lower | 29117 | 134 | 89 | 0 | 0 | 0 | - | deleted |
| Stage III | H25 | Normal | Lower | 26607 | 1840 | 1546 | 6 | 0 | 0 | - | deleted |
| Stage III | H26 | Normal | Lower | 31219 | 336 | 146 | 0 | 0 | 0 | - | deleted |
| Stage V | FH24 | Normal | upper | 30954 | 6306 | 6205 | 20 | 0 | 0 | - | deleted |
| Stage V | FH31 | Normal | Lower | 34728 | 1217 | 1181 | 3 | 0 | 0 | - | deleted |
| Stage V | FH32 | Normal | Lower | 35954 | 8240 | 8229 | 23 | 0 | 0 | - | deleted |
| Stage V | FH33 | Normal | Lower | 37793 | 1653 | 1595 | 4 | 0 | 0 | - | deleted |
| Stage V | FH34 | Normal | Lower | 31520 | 2364 | 2064 | 7 | 0 | 0 | - | deleted |
| Stage V | FH35 | Normal | Lower | 31547 | 1005 | 888 | 3 | 0 | 0 | - | deleted |
| Stage V | FH36 | Normal | Lower | 28225 | 520 | 454 | 2 | 0 | 0 | - | deleted |
| Stage VI | P26 | Normal | upper | 28060 | 5852 | 5814 | 21 | 0 | 0 | - | deleted |
| Stage VI | P31 | Normal | Lower | 27284 | 1111 | 966 | 4 | 0 | 0 | - | deleted |
| Stage VI | P32 | Normal | Lower | 27029 | 429 | 401 | 1 | 0 | 0 | - | deleted |
| Stage VI | P33 | Normal | Lower | 32851 | 2099 | 2065 | 6 | 0 | 0 | - | deleted |
| Stage VI | P34 | Normal | Lower | 27884 | 1274 | 1051 | 4 | 0 | 0 | - | deleted |
| Stage VI | P35 | Normal | Lower | 26446 | 4994 | 4758 | 18 | 0 | 0 | - | deleted |
| Stage VI | P36 | Normal | Lower | 26048 | 1611 | 1570 | 6 | 0 | 0 | - | deleted |
| Stage VI | WYD1 | no casing soil | upper | 60193 | 3541 | 3541 | 6 | 0 | 0 | - | deleted |
| Stage VI | WYD2 | no casing soil | upper | 57924 | 3409 | 3409 | 6 | 0 | 0 | - | deleted |
| Stage VI | WYD3 | no casing soil | upper | 48510 | 3276 | 3276 | 7 | 0 | 0 | - | deleted |
| Stage VI | WYD4 | no casing soil | upper | 59570 | 5077 | 5077 | 9 | 0 | 0 | - | deleted |
| Stage VI | WYD5 | no casing soil | upper | 68035 | 7371 | 7371 | 11 | 0 | 0 | - | deleted |
| Stage VI | WYD6 | no casing soil | upper | 66662 | 6774 | 6774 | 10 | 0 | 0 | - | deleted |
| Stage VI | WYU1 | no casing soil | Lower | 52158 | 3642 | 3642 | 7 | 0 | 0 | - | deleted |
| Stage VI | WYU2 | no casing soil | Lower | 71519 | 5571 | 5571 | 8 | 0 | 0 | - | deleted |
| Stage VI | WYU3 | no casing soil | Lower | 61300 | 3392 | 3392 | 6 | 0 | 0 | - | deleted |
| Stage VI | WYU4 | no casing soil | Lower | 52502 | 4845 | 4845 | 9 | 0 | 0 | - | deleted |
| Stage VI | WYU5 | no casing soil | Lower | 42231 | 3141 | 3141 | 7 | 0 | 0 | - | deleted |
| Stage VI | WYU6 | no casing soil | Lower | 72653 | 6365 | 6365 | 9 | 0 | 0 | - | deleted |
| Stage VII | F11 | Normal | fruit-body | 26656 | 407 | 350 | 1 | 0 | 0 | - | deleted |
| Stage VII | F12 | Normal | fruit-body | 28300 | 5537 | 1582 | 6 | 0 | 0 | - | deleted |
| Stage VII | F13 | Normal | fruit-body | 29116 | 146 | 126 | 0 | 0 | 0 | - | deleted |
| Stage VII | F14 | Normal | fruit-body | 26078 | 1120 | 572 | 2 | 0 | 0 | - | deleted |
| Stage VII | F15 | Normal | fruit-body | 31471 | 1561 | 286 | 1 | 0 | 0 | - | deleted |
| Stage VII | F16 | Normal | fruit-body | 29774 | 4638 | 4021 | 14 | 0 | 0 | - | deleted |
| Stage VII | F32 | Normal | upper | 30551 | 4017 | 3795 | 12 | 0 | 0 | - | deleted |
| Stage VII | F36 | Normal | upper | 31930 | 7346 | 7228 | 23 | 0 | 0 | - | deleted |
| Stage VII | F41 | Normal | Lower | 31876 | 928 | 892 | 3 | 0 | 0 | - | deleted |
| Stage VII | F42 | Normal | Lower | 32350 | 336 | 326 | 1 | 0 | 0 | - | deleted |
| Stage VII | F43 | Normal | Lower | 28981 | 545 | 256 | 1 | 0 | 0 | - | deleted |
| Stage VII | F44 | Normal | Lower | 35425 | 188 | 94 | 0 | 0 | 0 | - | deleted |
| Stage VII | F45 | Normal | Lower | 34929 | 1052 | 763 | 2 | 0 | 0 | - | deleted |
| Stage VII | WD1 | no casing soil | upper | 26720 | 2056 | 2056 | 8 | 0 | 0 | - | deleted |
| Stage VII | WD2 | no casing soil | upper | 29247 | 5225 | 5225 | 18 | 0 | 0 | - | deleted |
| Stage VII | WD3 | no casing soil | upper | 30011 | 4678 | 4678 | 16 | 0 | 0 | - | deleted |
| Stage VII | WD4 | no casing soil | upper | 30696 | 6322 | 6322 | 21 | 0 | 0 | - | deleted |
| Stage VII | WD5 | no casing soil | upper | 29767 | 2402 | 2402 | 8 | 0 | 0 | - | deleted |
| Stage VII | WD6 | no casing soil | upper | 27488 | 6682 | 6682 | 24 | 0 | 0 | - | deleted |
| Stage VII | WU1 | no casing soil | Lower | 29850 | 2378 | 2378 | 8 | 0 | 0 | - | deleted |
| Stage VII | WU2 | no casing soil | Lower | 26788 | 3347 | 3347 | 12 | 0 | 0 | - | deleted |
| Stage VII | WU3 | no casing soil | Lower | 28929 | 6204 | 6204 | 21 | 0 | 0 | - | deleted |
| Stage VII | WU4 | no casing soil | Lower | 32085 | 5560 | 5560 | 17 | 0 | 0 | - | deleted |
| Stage VII | WU5 | no casing soil | Lower | 30717 | 6615 | 6615 | 22 | 0 | 0 | - | deleted |
| Stage VII | WU6 | no casing soil | Lower | 29111 | 6384 | 6384 | 22 | 0 | 0 | - | deleted |
| Stage I | OS1 | Normal | soil | 32459 | 8589 | 8587 | 26 | 8587 | 26 | 8587 | Retained |
| Stage I | OS2 | Normal | soil | 28619 | 7100 | 7096 | 25 | 7096 | 25 | 7096 | Retained |
| Stage I | OS3 | Normal | soil | 31630 | 8615 | 8614 | 27 | 8614 | 27 | 8614 | Retained |
| Stage I | OS4 | Normal | soil | 53069 | 17858 | 17855 | 34 | 17855 | 34 | 17855 | Retained |
| Stage I | OS5 | Normal | soil | 34425 | 10672 | 10664 | 31 | 10664 | 31 | 10664 | Retained |
| Stage I | OS6 | Normal | soil | 38361 | 14034 | 14033 | 37 | 14033 | 37 | 14033 | Retained |
| Stage V | FH11 | Normal | casing soil | 28177 | 11598 | 11593 | 41 | 11593 | 41 | 11593 | Retained |
| Stage V | FH12 | Normal | casing soil | 25776 | 8475 | 8460 | 33 | 8460 | 33 | 8460 | Retained |
| Stage V | FH13 | Normal | casing soil | 25535 | 11457 | 11449 | 45 | 11449 | 45 | 11449 | Retained |
| Stage V | FH14 | Normal | casing soil | 24308 | 10229 | 10220 | 42 | 10220 | 42 | 10220 | Retained |
| Stage V | FH15 | Normal | casing soil | 25504 | 9148 | 9141 | 36 | 9141 | 36 | 9141 | Retained |
| Stage V | FH16 | Normal | casing soil | 25677 | 12751 | 12746 | 50 | 12746 | 50 | 12746 | Retained |
| Stage V | FH21 | Normal | upper | 27405 | 12179 | 12157 | 44 | 12157 | 44 | 12157 | Retained |
| Stage V | FH22 | Normal | upper | 29050 | 11241 | 11197 | 39 | 11197 | 39 | 11197 | Retained |
| Stage V | FH23 | Normal | upper | 28585 | 8912 | 8883 | 31 | 8883 | 31 | 8883 | Retained |
| Stage V | FH25 | Normal | upper | 36364 | 15516 | 15292 | 42 | 15292 | 42 | 15292 | Retained |
| Stage V | FH26 | Normal | upper | 30861 | 8278 | 8207 | 27 | 8207 | 27 | 8207 | Retained |
| Stage VI | P11 | Normal | casing soil | 31554 | 13609 | 13604 | 43 | 13604 | 43 | 13604 | Retained |
| Stage VI | P12 | Normal | casing soil | 31122 | 12514 | 12492 | 40 | 12492 | 40 | 12492 | Retained |
| Stage VI | P13 | Normal | casing soil | 28809 | 12560 | 12557 | 44 | 12557 | 44 | 12557 | Retained |
| Stage VI | P14 | Normal | casing soil | 30506 | 10348 | 10341 | 34 | 10341 | 34 | 10341 | Retained |
| Stage VI | P15 | Normal | casing soil | 26937 | 9287 | 9280 | 34 | 9280 | 34 | 9280 | Retained |
| Stage VI | P16 | Normal | casing soil | 31103 | 11694 | 11682 | 38 | 11682 | 38 | 11682 | Retained |
| Stage VI | P21 | Normal | upper | 36442 | 10250 | 10203 | 28 | 10203 | 28 | 10203 | Retained |
| Stage VI | P22 | Normal | upper | 29947 | 7889 | 7877 | 26 | 7877 | 26 | 7877 | Retained |
| Stage VI | P23 | Normal | upper | 26026 | 8853 | 8832 | 34 | 8832 | 34 | 8832 | Retained |
| Stage VI | P24 | Normal | upper | 28407 | 9640 | 9613 | 34 | 9613 | 34 | 9613 | Retained |
| Stage VI | P25 | Normal | upper | 28404 | 8695 | 8655 | 30 | 8655 | 30 | 8655 | Retained |
| Stage VII | F21 | Normal | casing soil | 33237 | 15191 | 15187 | 46 | 15187 | 46 | 15187 | Retained |
| Stage VII | F22 | Normal | casing soil | 33087 | 13921 | 13913 | 42 | 13913 | 42 | 13913 | Retained |
| Stage VII | F23 | Normal | casing soil | 25676 | 11654 | 11645 | 45 | 11645 | 45 | 11645 | Retained |
| Stage VII | F24 | Normal | casing soil | 28799 | 12029 | 12000 | 42 | 12000 | 42 | 12000 | Retained |
| Stage VII | F25 | Normal | casing soil | 28936 | 12715 | 12696 | 44 | 12696 | 44 | 12696 | Retained |
| Stage VII | F26 | Normal | casing soil | 29214 | 12227 | 12215 | 42 | 12215 | 42 | 12215 | Retained |
| Stage VII | F31 | Normal | upper | 40708 | 12526 | 12492 | 31 | 12492 | 31 | 12492 | Retained |
| Stage VII | F33 | Normal | upper | 28420 | 8621 | 8596 | 30 | 8596 | 30 | 8596 | Retained |
| Stage VII | F34 | Normal | upper | 36109 | 10940 | 10923 | 30 | 10923 | 30 | 10923 | Retained |
| Stage VII | F35 | Normal | upper | 45887 | 13213 | 13203 | 29 | 13203 | 29 | 13203 | Retained |
| Stage V | SF1 | Sterilized casing soil | casing soil | 31771 | 133 | 133 | 0 | 0 | 0 | - | deleted |
| Stage V | SF2 | Sterilized casing soil | casing soil | 30781 | 375 | 375 | 1 | 0 | 0 | - | deleted |
| Stage V | SF3 | Sterilized casing soil | casing soil | 31729 | 342 | 342 | 1 | 0 | 0 | - | deleted |
| Stage V | SF4 | Sterilized casing soil | casing soil | 32864 | 285 | 285 | 1 | 0 | 0 | - | deleted |
| Stage V | SF5 | Sterilized casing soil | casing soil | 27347 | 566 | 566 | 2 | 0 | 0 | - | deleted |
| Stage V | SF6 | Sterilized casing soil | casing soil | 30579 | 557 | 557 | 2 | 0 | 0 | - | deleted |
| Stage V | SFD1 | Sterilized casing soil | Lower | 27286 | 248 | 192 | 1 | 0 | 0 | - | deleted |
| Stage V | SFD2 | Sterilized casing soil | Lower | 27158 | 88 | 69 | 0 | 0 | 0 | - | deleted |
| Stage V | SFD3 | Sterilized casing soil | Lower | 27899 | 274 | 243 | 1 | 0 | 0 | - | deleted |
| Stage V | SFD4 | Sterilized casing soil | Lower | 36763 | 207 | 188 | 1 | 0 | 0 | - | deleted |
| Stage V | SFD5 | Sterilized casing soil | Lower | 29681 | 109 | 99 | 0 | 0 | 0 | - | deleted |
| Stage V | SFD6 | Sterilized casing soil | Lower | 38991 | 84 | 65 | 0 | 0 | 0 | - | deleted |
| Stage V | SFU1 | Sterilized casing soil | upper | 29553 | 5431 | 3935 | 13 | 0 | 0 | - | deleted |
| Stage V | SFU2 | Sterilized casing soil | upper | 36967 | 9554 | 7337 | 20 | 0 | 0 | - | deleted |
| Stage V | SFU3 | Sterilized casing soil | upper | 30993 | 6099 | 4827 | 16 | 0 | 0 | - | deleted |
| Stage V | SFU4 | Sterilized casing soil | upper | 28483 | 324 | 183 | 1 | 0 | 0 | - | deleted |
| Stage V | SFU5 | Sterilized casing soil | upper | 39446 | 1368 | 773 | 2 | 0 | 0 | - | deleted |
| Stage V | SFU6 | Sterilized casing soil | upper | 29463 | 1150 | 760 | 3 | 0 | 0 | - | deleted |
| Stage VI | SPCS1 | Sterilized casing soil | casing soil | 30988 | 121 | 121 | 0 | 0 | 0 | - | deleted |
| Stage VI | SPCS2 | Sterilized casing soil | casing soil | 31226 | 376 | 376 | 1 | 0 | 0 | - | deleted |
| Stage VI | SPCS3 | Sterilized casing soil | casing soil | 32157 | 208 | 208 | 1 | 0 | 0 | - | deleted |
| Stage VI | SPCS4 | Sterilized casing soil | casing soil | 34398 | 3128 | 3128 | 9 | 0 | 0 | - | deleted |
| Stage VI | SPCS5 | Sterilized casing soil | casing soil | 35303 | 1837 | 1837 | 5 | 0 | 0 | - | deleted |
| Stage VI | SPCS6 | Sterilized casing soil | casing soil | 29670 | 6760 | 6760 | 23 | 0 | 0 | - | deleted |
| Stage VI | SPD1 | Sterilized casing soil | Lower | 31429 | 3298 | 1063 | 3 | 0 | 0 | - | deleted |
| Stage VI | SPD2 | Sterilized casing soil | Lower | 27825 | 5954 | 5054 | 18 | 0 | 0 | - | deleted |
| Stage VI | SPD3 | Sterilized casing soil | Lower | 33870 | 615 | 290 | 1 | 0 | 0 | - | deleted |
| Stage VI | SPD4 | Sterilized casing soil | Lower | 29091 | 178 | 76 | 0 | 0 | 0 | - | deleted |
| Stage VI | SPD5 | Sterilized casing soil | Lower | 29198 | 3305 | 2221 | 8 | 0 | 0 | - | deleted |
| Stage VI | SPD6 | Sterilized casing soil | Lower | 34577 | 381 | 159 | 0 | 0 | 0 | - | deleted |
| Stage VI | SPU1 | Sterilized casing soil | upper | 32864 | 1892 | 1373 | 4 | 0 | 0 | - | deleted |
| Stage VI | SPU2 | Sterilized casing soil | upper | 31783 | 7310 | 5688 | 18 | 0 | 0 | - | deleted |
| Stage VI | SPU3 | Sterilized casing soil | upper | 34021 | 1974 | 1350 | 4 | 0 | 0 | - | deleted |
| Stage VI | SPU4 | Sterilized casing soil | upper | 29333 | 387 | 197 | 1 | 0 | 0 | - | deleted |
| Stage VI | SPU5 | Sterilized casing soil | upper | 31727 | 1958 | 1266 | 4 | 0 | 0 | - | deleted |
| Stage VI | SPU6 | Sterilized casing soil | upper | 36688 | 3717 | 1956 | 5 | 0 | 0 | - | deleted |


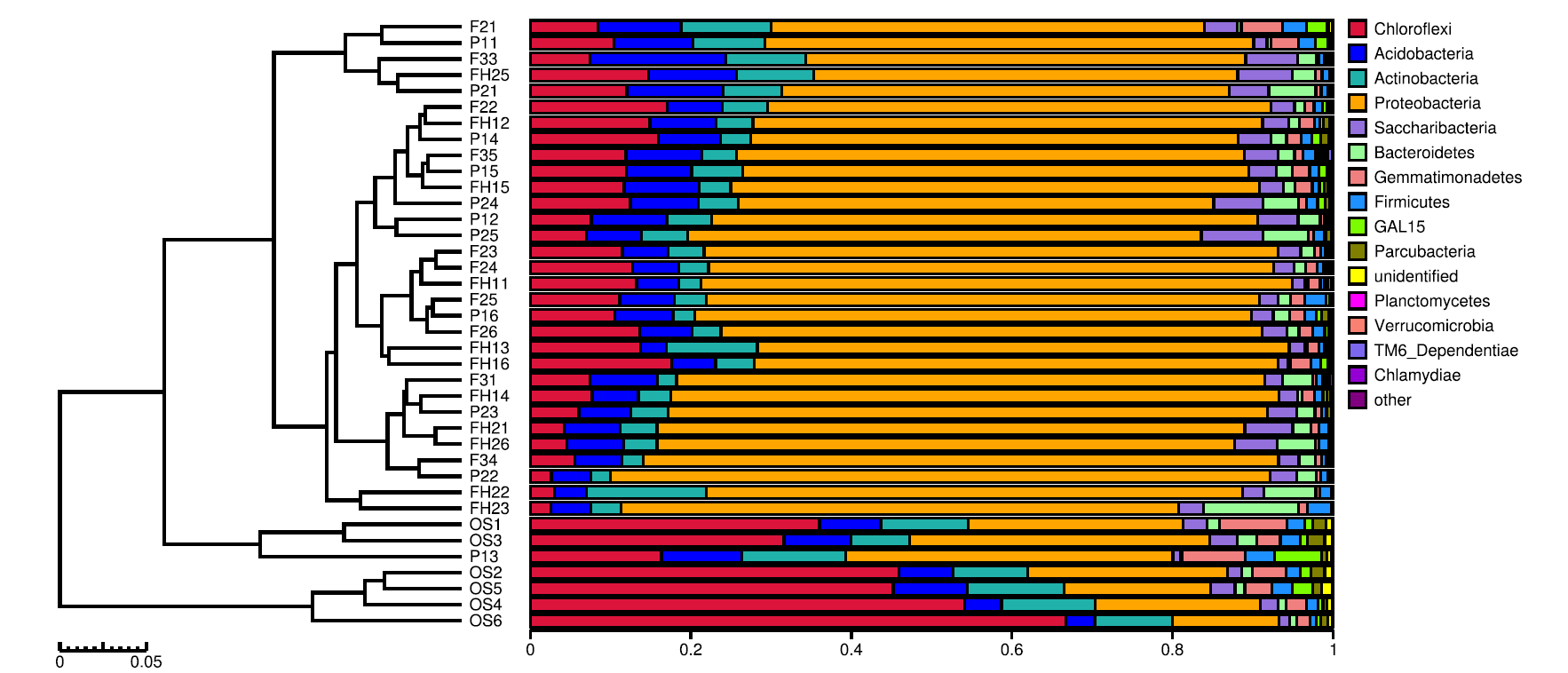


FIGURE S1. The relative abundance of bacteria in different samples at phylum level.


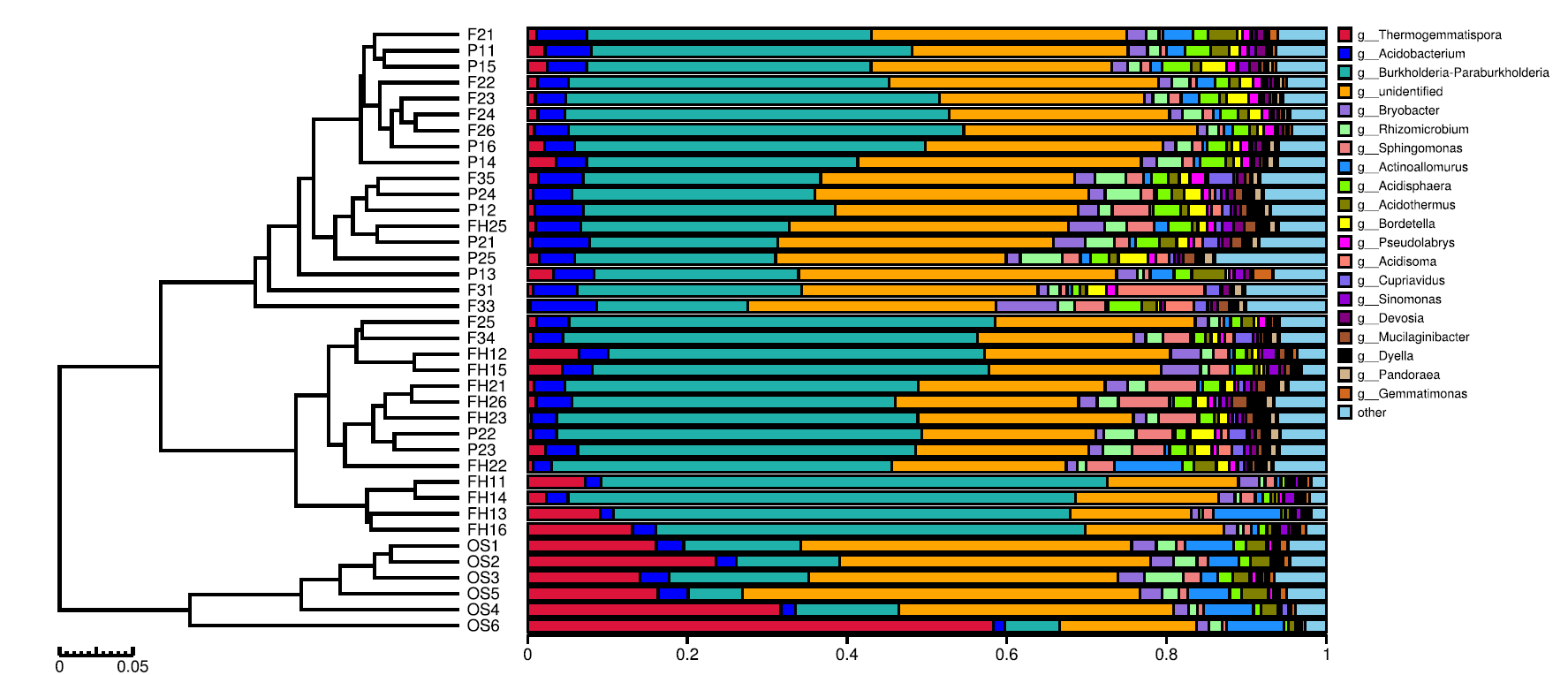


FIGURE S2. The relative abundance of bacteria in different samples at genus level.
